# Dry Powder Inhalation for Lung Delivery in Cystic Fibrosis

**DOI:** 10.3390/pharmaceutics15051488

**Published:** 2023-05-13

**Authors:** Xiaoxuan Han, Danni Li, Felisa Reyes-Ortega, Elena K. Schneider-Futschik

**Affiliations:** Department of Biochemistry and Pharmacology, School of Biomedical Sciences, Faculty of Medicine, Dentistry and Health Sciences, The University of Melbourne, Parkville, VIC 3010, Australia

**Keywords:** cystic fibrosis, dry powder formulation, clinical factors, pulmonary medicines, respiratory delivery

## Abstract

Pulmonary drug delivery has long been used for local and systemic administration of different medications used in acute and chronic respiratory diseases. Certain lung diseases, such as cystic fibrosis, rely heavily on chronic treatments, including targeted lung delivery. Pulmonary drug delivery possesses various physiological advantages compared to other delivery methods and is also convenient for the patient to use. However, the formulation of dry powder for pulmonary delivery proves challenging due to aerodynamic restrictions and the lower tolerance of the lung. The aim of this review is to provide an overview of the respiratory tract structure in patients with cystic fibrosis, including during acute and chronic lung infections and exacerbations. Furthermore, this review discusses the advantages of targeted lung delivery, including the physicochemical properties of dry powder and factors affecting clinical efficacy. Current inhalable drug treatments and drugs currently under development will also be discussed.

## 1. Introduction

### 1.1. Respiratory Tract

The human respiratory tract is responsible for the exchange of oxygen and carbon dioxide, and it is composed of the upper respiratory tract (URT) and the lower respiratory tract (LRT). The URT includes the nasal cavity, pharynx, and larynx, whereas the LRT includes the trachea, bronchi, and lungs [1,2]. Lungs are where gas exchange takes place, and the anatomical structure of them is bifurcated into the right and left lung, while the left lung and right lung are divided into two and three lobes, respectively. Further down the lungs, air passages are divided into tiny sacs called alveoli [1,2].

Administering drugs directly to the lung is an ideal way of treating both local and systemic (affecting more than one organ system) types of lung diseases, as it offers many considerable advantages over the conventional route of administration. To facilitate ideal gas exchange, the adult human airway surface area is approximately 70 m^2^ [3]. This means drugs administered directly to the lungs are rapidly absorbed due to the thin epithelial membrane (0.1–0.2 mm), a large surface area, and low levels of enzymatic activity, enabling a rapid onset of therapeutic action [1,2,4,5,6]. Additionally, pulmonary drug delivery can avoid the tricky first-pass metabolism that directly delivers active pharmaceutical ingredients (APIs) to the lungs, reducing off-target side effects [7,8].

On the other hand, pulmonary drug delivery proves to be very challenging due to drug formulation instabilities due to interaction forces between the particles, properties of the drug like particle size and solubility, poor delivery efficiency due to the removal of inhaled drug particles via mucociliary clearance, exhalation of drug particles, and phagocytosis by pulmonary macrophages [4,6]. Several approaches have been used to overcome these obstacles and improve the bioavailability of administered drugs.

### 1.2. Cystic Fibrosis

Cystic fibrosis is a multisystem autosomal recessive disease that is caused by mutations in the cystic fibrosis transmembrane conductance regulator (CFTR) gene, resulting in a dysfunctional CFTR protein [9]. Its main function is to regulate chlorine transport [9]. Additionally, the CFTR protein regulates bicarbonate and sodium transport [10]. In CF patients, depending on the mutation, the CFTR gene is either dysfunctional (e.g., class 3 mutations such as the G551D CFTR mutation) or fully absent (e.g., class 2 mutations such as the F508del CFTR mutation). This results in the accumulation of chloride ions within the cell and more sodium ions being actively transported into the cells. This alters cellular osmotic pressure, increasing the movement of water into the cell. This leads to thicker and dehydrated mucus in the airway, resulting in impaired mucus clearance and decreased airflow [11].

### 1.3. Understanding Dry Powder Inhalers

Inhalable dry powder (IDP) therapy is a commonly used method for delivering drugs to the lungs for the treatment of respiratory conditions including cystic fibrosis, chronic obstructive pulmonary disease, asthma, and lung infections [12]. Recently, IDP has gained increasing interest in the treatment of CF, such as in the treatment of lung infections. IDP administration offers several advantages over traditional oral and intravenous medications, including improved bioavailability, reduced systemic side effects, and improved compliance [7,13]. A promising new approach is also the potential lung delivery of novel CF modulators such as ivacaftor, lumacaftor, tezacaftor, and elexacaftor.

There are various types of DPIs on the market, such as podhaler, turbuhaler, accuhaler, handihaler, breezhaler, etc. [14]. Inhaler devices are classified based on their mode of delivery. For example, reservoir-based powder inhalers such as turbohalers contain a substantial amount of medication that can last for several weeks [15]. On the other hand, capsule-based inhalers dispense a single dose of a drug. These inhalers can be filled either with a single capsule or contain a blister strip that is dispensed sequentially from one application to the next [15]. One example of DPI used in CF treatment is the T-326, also known as the Podhaler device; which is used with Tobramycin inhalation powder. This device is a capsule-based single-unit dose inhaler; it works by pressing a plunger on the bottom end of the device, followed by inhalation [14].

### 1.4. Physicochemical Properties of Dry Powder

In the evaluation of dry powder therapy, its physicochemical properties, such as size, shape, density, and surface electrostatic charge, play a crucial role in its drug delivery and efficacy. For example, small particles tend to stick together and form agglomerates due to adhesive forces [16].

#### 1.4.1. Size

Particle size is one of the most important factors that directly influences the drug’s deposition in the lungs and, thereby, its efficacy. In order to evaluate effective dry powder formulations, a certain drug particle size is required to enable them to reach deep enough into the lungs. Studies have shown that the most appropriate particle size for best performance is a diameter between 1 and 5 μm [16,17,18]. Particles smaller than 1 μm are too cohesive, and particles greater than 5 μm are less cohesive, meaning they do not reach their target destination in high enough concentrations [4,17,19].

#### 1.4.2. Shape and Surface Morphology

Particle shape and morphology are shown to influence drug particle flowability, aerosolization, and deposition performance in the lungs [20]. Generally, particles with complex shapes and rough surfaces have a lower contact area and lower van der Waals forces compared to those with regular shapes. Consequently, they are less likely to form aggregates [4,21]. Moreover, Hassan and Lau investigated the shape effects of dry particles on flowability, aerosolization, and deposition properties by comparing similar-sized particles of different shapes, such as spheres, needles, pollen, and cubes. They show that the pollen-shaped particles exhibit the best flowability and highest emitted dose, among others, which proves the significant impact of particle shape on dry powder formulation [20].

**Figure 1:**
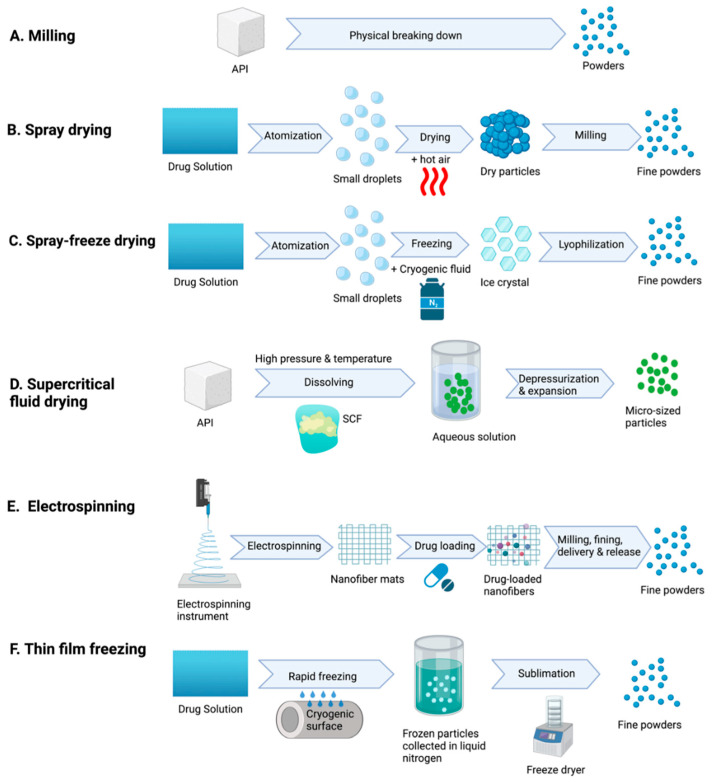
Current techniques for making dry powder drug particles. (**A**) Milling: large APIs will be mechanically broken down into small particles. (**B**) Spray drying: the drug solution will be atomized into small droplets and then dried into particles. Further milling can be applied to shape the size. (**C**) Spray-freeze drying: small drug droplets from atomization will be rapidly frozen in cryogenic fluid such as liquid nitrogen. Small crystals will then be lyophilized to remove the water inside. (**D**) Supercritical fluid drying: APIs will be dissolved in supercritical fluid (SCF), a mixture of liquid and gas under high pressure and temperature. Depressurization and expansion will remove solvents, allowing the formation of micro-sized particles. (**E**) Electrospinning: nanofiber mats generated via electrospinning can be applied to load drugs and refine them into suitable sizes for DPIs. (**F**) Thin film freezing: drug solution will be rapidly frozen on the cryogenic surface, and frozen particles will be collected in liquid nitrogen. Sublimation via a freeze dryer will then remove the solvent to generate fine powders.

Prior to inhaler device development, different techniques are utilized to make the dry powder drug particles with desired properties, such as size, shape, density, and surface electronic charges; current techniques mainly include milling, spray drying, spray freeze drying, supercritical fluid drying, and electrospinning (Figure 1).

Milling is a technique used to modify particle size by physically breaking down large particles into smaller ones [22]. Apart from size, the milling technique also changes the particles’ shape and surface roughness unpredictably, potentially influencing the deposition properties of drug particles, which is a potential concern for this technique [22].

Spray drying was developed at the end of the 1800s and is now a commonly used technique for dry powder preparation for inhalation. It typically involves several steps, including the atomization of the drug solution into small droplets. These droplets are rapidly dried using hot air and collected using a specific device. The collected sample may then undergo further processing, such as milling, to achieve the desired property [4,23,24,25]. This technique offers several advantages over others such as milling and freeze-drying, as it is faster and easier to produce particles with controlled properties such as size and surface roughness [24,25].

Spray-freeze drying is a relatively new method that combines spray drying and freeze drying techniques that are utilized for DPI drug formation, but at a higher cost [25]. In the spray-freeze drying process, drug solution is first atomized into small droplets and then rapidly frozen using a cryogenic fluid such as liquid nitrogen. Liquid nitrogen is then lyophilized to remove the ice and produce dry powder [4,25].

Supercritical fluid drying is another technique used to prepare micro-sized particles for inhalation that has attracted much attention in the last few decades [25,26,27]. Supercritical fluid is the thermodynamic condition for chemical substances. When the substance is heated and pressurized beyond its critical point, it will have the properties of both a liquid and a gas [25,27]. Supercritical fluid drying has several advantages, including the ability to produce particles with a narrow size distribution and high purity and flow properties [25].

Electrospinning is an electrodynamic process that involves the creation of ultra-fine fibers by using a high electrical charge to draw a polymer solution or melt polymer [28]. This technique has gained significant attention in recent years due to its ability to create materials with unique properties that can be beneficial in DPI formulation. For example, the electrospun fibers that were created by electrospinning showed outstanding properties, such as a large surface area and tenable surface morphologies [29]. In addition, cryo-milled polyvinyl alcohol nanofiber mats loaded with α-chymotrypsin by electrospinning have been shown to have suitable inhalation properties for DPI utilization, where they are porous to fine particles with adjustable particle size [30]. This novel method may be suitable for the DPI formulation as it avoids heated steps in the process [30].

Thin film freezing (TFF) is a new emerging ultra-rapid freeze-drying technique that is used to produce powder drug particles. In the TFF process, the API and stabilizer solution are rapidly frozen on a cryogenically frozen surface, followed by the collection of frozen particles and the removal of the solvent through sublimation in a freeze-dryer [31,32]. This freezing technology has been used to improve the physical properties of the drug particles as it converts the crystalline drug into an amorphous form through supercooling; the dry powder obtained through the TFF process is usually a fluffy, porous, brittle matrix, which often exhibits superior aerosol performance properties [31,33]. As a result, it is an ideal option for DPI particle formulation.

In summary, it was shown that all the presented methods allow people to adjust particle physicochemical properties such as size, shape, and morphology, thereby optimizing the formulation of DPI.

## 2. Drug Administration

Drug administration directly to the lung is achieved by aerosolization, which refers to a suspension of fine solid particles or liquid droplets in a gas [34]. DPI therapy is a method of delivering APIs directly to the lungs by inhaling through specific devices. Pressurized metered-dose inhalers, nebulizers, and dry powder inhalers are the three main types of devices for pulmonary medication delivery; however, the dry powder inhaler was considered a better option since it requires lower standard patient coordination as it is breath-activated, and in addition to its better lung deposition property, it is also more environmentally friendly as it produces fewer chlorofluorocarbons [8,35,36,37,38].

### 2.1. Administration and Clearance

**Figure 2:**
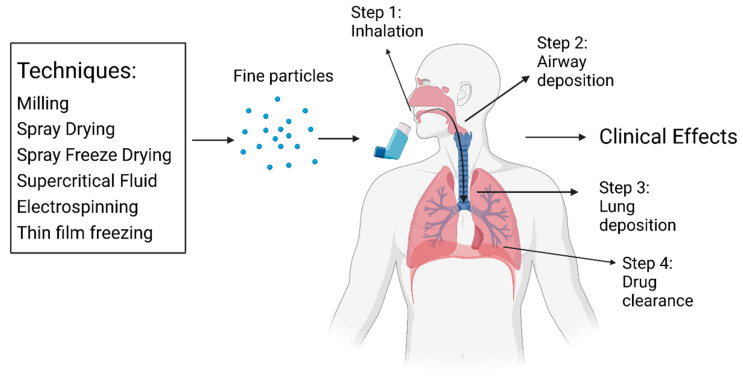
Simplified illustration of DPI formulation and mechanism of action.

The administration of a DPI correctly involves four steps. Firstly, in order to deliver an active pharmaceutical ingredient [39] to the respiratory tract, the patient must inhale through the DPI device, which provides energy to break up the consolidated drug powder. Then the drug powder particles are carried by lactose, which is commonly added as a dry powder carrier [40,41,42]. Next, drug particles will be deposited in the airways, where they can reach the targeted site of action. Small particles are able to reach the deeper areas of the lungs, while larger particles are more likely to deposit in the upper airway (Figure 2). In the last step, the remaining powder will either be removed by exhalation or by native lung clearance mechanisms (mucociliary clearance, macrophage clearance) [8,36,43].

### 2.2. Advantages and Limitations

DPI has numerous advantages in comparison with other routes of administration. The most striking one is the avoidance of the first-pass metabolism effects, which directly deliver the APIs to the lungs, reducing off-target side effects [7,8]. DPI also allows for more rapid absorption of APIs into the bloodstream, allowing for fast onsite action [8,44]. Lastly, DPI is more flexible and easier to use, and it provides needle-free medication, which is often preferred by patients and largely improves compliance.

However, there are limitations that restrict the use of DPIs in some cases. Firstly, DPIs require patients’ forceful inspiration in order to generate enough air flow rate to deliver the drug particles deep enough into the lung and perform their function properly. Unfortunately, in many respiratory diseases, the patient’s lung function is reduced, leading to insufficient air flow, which reduces drug exposure in the lung [45]. Secondly, the DPIs require proper handling and storage in order to perform well. For example, according to Sandler et al., a study found that only slightly more than one-third of patients who had not previously used an inhaler were able to use it properly without any guidance; however, with proper training from a healthcare provider, over 95% of participants were able to acquire the essential technical skills to effectively use the inhaler [36,46]. Thirdly, the performance of DPIs is influenced by a slight change in air humidity, which leads to reduced stability of the drugs in the inhaler and hence affects clinical outcomes [47].

## 3. Factors Affecting Clinical Efficacy

**Figure 3:**
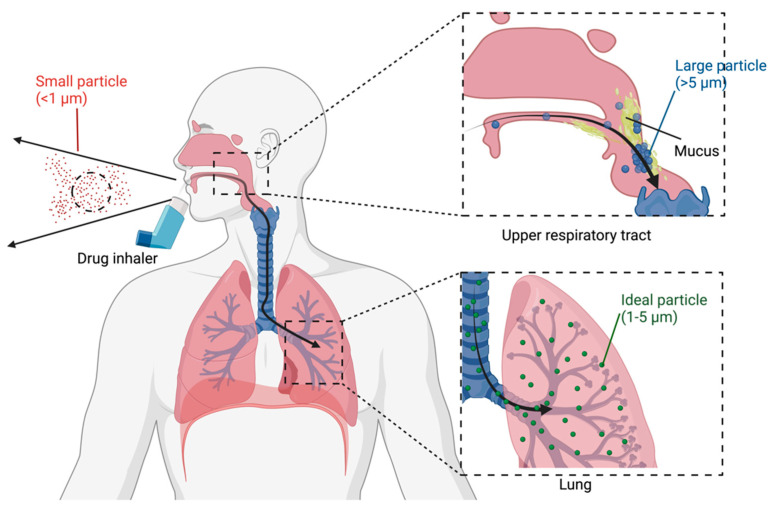
Comparing the size of DPI. Small-sized drugs (<1 μm) are likely to be exhaled at the start and are unable to reach the respiratory tract. Large-sized drugs (>5 μm) will be trapped and deposited in the upper respiratory tract without reaching the lung. The optimal size of the inhaled dry powder is 1–5 μm.

The clinical efficacy and outcome of DPI depend on various factors, but these three play a major role: the dry powder particle formulation, the DPI device design, and the patients’ inhalation technique or compliance. Firstly, the physicochemical properties such as particle size, shape, and density extensively influence the DPI performance, as they affect the dry powder deposition and absorption of the drug in the lungs (Figure 3).

To ensure efficient absorption and clinical effects, particle size optimization is crucial, as small particles may be quickly exhaled, while those larger than 5 μm may be deposited in the upper respiratory tract without penetrating the mucus and reaching the lungs [16,17,18].

DPI devices also influence the clinical efficacy of drugs; there are various types on the market, and their working mechanism is mostly the same, which depends on patients’ inspiration, but can vary in a few properties, such as resistance and airflow rate. Some devices contain carrier particles such as lactose, which improve the flowability of the powder. Levy et al. analyzed the properties of multiple inhalers from other reviews to determine the importance of selecting the appropriate device for different patients [36]. Moreover, Haikarainen et al. compared the in vitro drug delivery characteristics of budesonide/formoterol Easyhaler^®^ and budesonide/formoterol Turbuhaler^®^, and they proved that Easyhaler^®^ performed better under different conditions, such as different air flow rates [48].

Lastly, the clinical efficacy of DPIs is also influenced by the patient’s pulmonary condition, sex, age, and education level [49,50,51]. Since DPI has a “passive” property, it requires correct technique and forceful air inflow to generate enough energy to de-aggregate the formulation. A study performed by Melani et al. showed that among 2288 records, the critical mistakes in the use of DPI were strongly associated with older age, lack of education, and lack of training on inhaler technique [52]. Therefore, inhaler technique training is necessary to minimize the potential errors. In addition, it has been proven that patients with lung dysfunctional diseases such as asthma have more difficulties using DPIs; therefore, the prescriber should consider the patient’s pulmonary conditions before prescribing DPIs [4,39].

## 4. Bacterial Infection in CF

Chronic bacterial colonization/infection is a hallmark of CF pathogenesis and is correlated with rapid decline of lung function and elevated morbidity and mortality in CF patients [53,54]. Mucus accumulation in the lung promotes an environment conducive to bacterial infections such as *Pseudomonas aeruginosa* (*P. aeruginosa*), *Haemophilus influenzae*, *Burkholderia cepacian*, *Burkholderia pseudomallei*, *Stenotrophomonas maltophilia*, *Klebsiella pneumoniae,* and *Acinetobacter baumannii* [55,56]. Overall, *P. aeruginosa* dominates CF pulmonary infections among adult patients, and it is often the most problematic to treat, as once chronic infection is established, the infecting strain becomes increasingly resistant to antibiotics [53]. Persistent *P. aeruginosa* infection is mainly due to biofilm-growing mucoid strains [57,58,59]. In addition, as mucociliary clearance in CF is dysregulated, chronic bacterial colonization leads to an exacerbated pro-inflammatory immune response [60,61].

## 5. Cystic Fibrosis Treatments

Currently, there is no cure for CF, and patients rely on lifelong treatments to slow down the progression of the disease. There are several treatments available depending on the disease severity and organ manifestation [62,63]. For example, inhaled hypertonic saline solution is used to hydrate the airway, decreasing mucus viscosity while increasing mucociliary clearance, thereby improving the patients’ lung function [62].

### 5.1. CFTR Modulators

CFTR modulators represent a pivotal progression in CF treatment because they are the only treatments that target the cause of the disease [10,64]. Three types of CFTR modulators exist: the potentiators, which can bind to CFTR and improve its function; the CFTR correctors, which increase the trafficking of CFTR protein to the cell membrane; and the CFTR amplifiers, which increase CFTR production [65]. Intrinsically, CFTR modulators improve CF patients’ lung function and reduce pulmonary exacerbations by increasing CFTR performance. In addition, Ivacaftor is the first and only clinically approved CFTR potentiator. It functions by binding to the CFTR protein, increasing its opening time and the transport of chloride ions, thereby restoring the CFTRs’ function [65]. It has been demonstrated to improve lung function significantly and improve mucociliary clearance [66]. Ivacaftor monotherapy is approved for class 3 mutations, including gating mutations such as G551D mutations (~2–3% of patients) and rare gating mutations such as G1244E, S1251N, S1255P, G1349D, G178R, G551S, S549N, S1252N, S549R, and R117H [67]. These mutations result in sufficient CFTR protein on the cell surface but impaired function [65]. In clinical trials, ivacaftor monotherapy improved lung function in patients with this mutation by 6.7% on average [66]. Furthermore, it has been shown to correct neutrophil degranulation, thereby increasing *P. aeruginosa* killing [68].

Ivacaftor forms part of all other CFTR modulator regimens with either lumacaftor, tezacaftor, or elexacaftor-tezacaftor. The ivacaftor-lumacaftor combination increases CFTR function by restoring the F508del-CFTR folding, and it has been shown to improve lung function and lead to a reduction in pulmonary exacerbations among CF patients that carry homozygous F508del-CFTR mutations, while there are no significant effects in patients carrying heterozygous mutations [67,69].

Tezacaftor-ivacaftor is approved for CF patients with a homozygous F508del CFTR mutation. Like lumacaftor, tezacaftor is a CFTR corrector and increases the CFTR protein’s transport to the cell membrane [70]. While tezacaftor corrects CFTR protein folding and presentation on the cell surface, ivacaftor additionally improves the CFTR opening duration, therefore reconstructing the ion transport and balancing the concentration. Consequently, less water moves into the cell, improving mucus clearance [71]. Tezacaftor-ivacaftor combination therapy increased the FEV1 of CF patients who have a homozygous F508del CFTR mutation by 4% after 24 weeks of administration compared to the placebo group. It has also demonstrated a 6.8% improvement in patients with a heterozygous F508del CFTR mutation compared to the placebo group after 4 and 8 weeks of administration [72].

Elexacaftor-tezacaftor-ivacaftor is a recently approved triple combination therapy for treating CF patients with one or two F508del CFTR mutations. It contains two correctors and one potentiator [73]. Elexacaftor-tezacaftor-ivacaftor resulted in a 14.3% improvement in FEV1 and a 63% lower pulmonary exacerbation rate than in the placebo group after 24 weeks of administration [74]. Triple combination therapy also showed greater performance in reducing sweat chloride concentration, which means better performance in fixing the CFTR ion transportation function in comparison with tezacaftor/ivacaftor therapy among CF patients with an F508del CFTR mutation [73].

### 5.2. Antibiotics

Antibiotics are commonly used to treat chronic and recurring infections in CF. There is increasing evidence that antibiotic therapy, such as inhaled tobramycin, can eradicate the early onset of *P. aeruginosa* infection [58]. In addition to treatments that target the lung, treatment of symptoms in other organs to relieve symptoms includes pancreatic enzymes to improve pancreatic insufficiency [75] in the gastrointestinal tract. Additionally, physical training has proven to be effective for patients to regulate disease progression [76].

### 5.3. Anti-Inflammatories

Managing the inflammatory condition in CF is crucial to preventing pulmonary exacerbation, as it is the primary process involved in this pathology. Various steps of the pathophysiological cascade can be targeted for treatment. In the past, studies on anti-inflammation have primarily focused on corticosteroids and nonsteroidal anti-inflammatory drugs such as ibuprofen [77]. Ibuprofen is recommended for preventing the loss of lung function in patients with a forced expiratory volume in 1 s (FEV1) greater than 60% predicted by inhibiting the pro-inflammatory enzyme cyclooxygenase, and it is also recommended for long-term treatment for CF airway inflammation [75]. Ibuprofen has also been shown to slow the progression of lung disease and reduce pulmonary exacerbations [75].

## 6. Dry Powder Inhalers in CF Therapy

**Figure 4:**
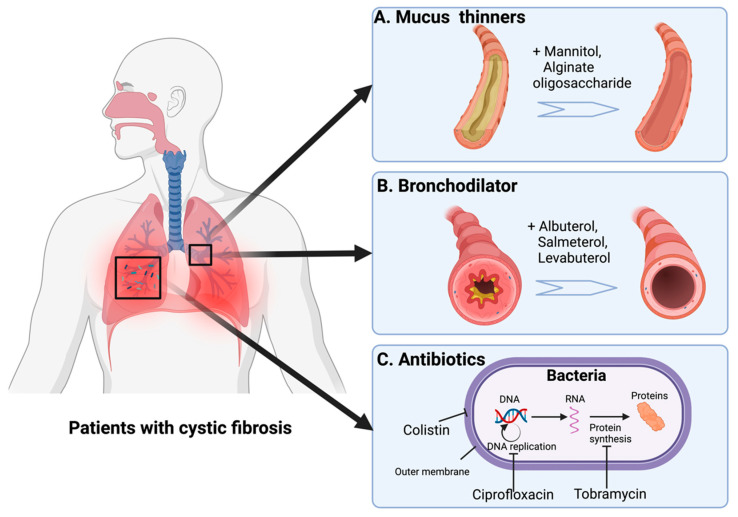
DPIs in treating patients with cystic fibrosis: mechanisms of action. Typical pulmonary manifestations in cystic fibrosis include thickened mucus, obstructed airways, and bacterial infection. DPIs developed to target those symptoms include: (**A**) Mucus thinners (mannitol and alginate oligosaccharides) to hydrate the airways; (**B**) bronchodilators (albuterol, salmeterol, and levabuterol) to relax airway muscle and then increase the airway lumen; and (**C**) antibiotics (tobramycin, colistin, and ciprofloxacin) to kill bacteria by disrupting different bacterial structure and mechanisms.

Currently, the DPI used in CF treatment includes mucus thinners, bronchodilators, and antibiotics (Table 1, Figure 4).

### 6.1. Mucus Thinners

The impairment of mucus clearance in the lower airway contributes to mucus accumulation. There are various types of mucolytics that can improve mucus clearance by optimizing the viscoelasticity of mucus, such as hypertonic saline, dornase alfa, alginate oligosaccharide, and mannitol [78,84].

**Figure 5:**
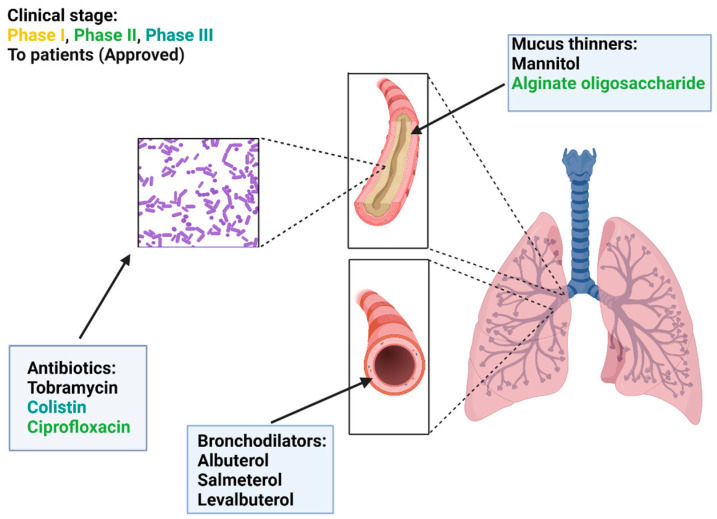
A schematic of cystic fibrosis lung and airway and colored DPI treatment options, which were divided into three categories: mucus thinners, antibiotics, and bronchodilators; they are colored based on their developmental stage. Yellow represents the phase I clinical trial, green represents the phase II clinical trial, blue represents the phase III clinical stage, and black represents approval by the FDA and EMA, respectively.

Due to their physicochemical properties, only mannitol and alginate oligosaccharides are administered as DPIs (Figure 5), while the others are administered as nebulizers [78,84,85,86]. Inhaled mannitol (400 mg) is administered twice a day. It improves mucus hydration via changing osmotic pressure and drawing water into the airway, thereby enhancing mucociliary clearance in CF patients [84]. The adverse effects of mannitol include coughing, headaches, throat irritation, and nausea [84].

### 6.2. Bronchodilators

Inhaled bronchodilators are usually administered prior to the mucus being thinner, with the general idea being that thinner mucus allows other medications to reach deeper into the lungs. Typically, inhaled bronchodilators in CF treatment are short-acting beta-agonists (SABAs) such as albuterol and levalbuterol, which bind to beta2-cholinergic receptors in the airway and trigger the activation of receptors, which helps the airway’s smooth muscle relaxation (Figure 5) [87]. They are commonly administered via metered-dose inhalers but may also be delivered through a DPI or nebulizer. Common side effects of SABAs include tremors, nausea, and vomiting.

### 6.3. Antibiotics

Inhaled dry powder antibiotics like tobramycin and colistin have been proven to be effective in treating CF patients with *P. aeruginosa* infections [88,89,90]. Tobramycin and colistin (Figure 5) are two types of antibiotics used to treat bacterial infections; they work differently. Tobramycin is a type of aminoglycoside that inhibits protein synthesis via binding the 16s ribosomal RNA of the bacterial 30s ribosomal unit, whereas colistin works by targeting the bacterial cell membrane, disrupting the cell envelope, and causing bacterial lysis [91,92,93]. Ciprofloxacin is also approved against lower respiratory tract infections; it kills bacteria by inhibiting DNA replication by targeting the bacterial DNA topoisomerase and DNA-gyrase [94]. The most common administration routes of ciprofloxacin are oral and intravenous administration; however, Stass et al. showed that inhaled ciprofloxacin reduced the systemic off-target effects compared to oral and intravenous administration [95].

## 7. Case Studies of the Effectiveness of DPIs on CF

Evidence of the effectiveness of DPIs in treating CF can be found in many previous studies. Different research groups have conducted clinical trials to demonstrate the safety and efficacy of various DPIs for treating CF. For example, DPI mannitol, a hyperosmotic agent that is used to improve CF patients’ mucus clearance, was examined in various studies to evaluate its efficacy and safety. A clinical trial with 423 subjects was performed to test the effectiveness of IDP mannitol in adults with CF, and results indicated a significant improvement in FEV1 compared with the control group. Another study proved that DPI mannitol was also efficacious and well tolerated in treating children with CF, as the results have shown significant improvements in lung function and sputum weight [96].

A study of inhaled dry powder mannitol on 324 CF patients also showed a similar result, but different from the other studies shown above, this study not only showed an intervention group that had a significant improvement on FEV1, but also a reduction in the incidence of pulmonary exacerbation by 35.4% compared to the control group, which was not mentioned in the other studies and was recommended by the European Medicines Agency to be considered in a clinical study [38,97].

In 2013, Galeva et al. used a double-blind clinical trial with 100 patients to prove that inhaled tobramycin powder, a class of antibiotic medication that significantly reduced bacterial sputum density measured from *P. aeruginosa*-infected CF patients in comparison to the placebo, also improved patients forced expiratory volume per second (FEV1) by 5.9%. This indicated that tobramycin inhalation powder was effective in treating CF with *P. aeruginosa* infection [98]. Similar results had also been elucidated by another study that contained 553 patients [99]. Furthermore, Tappenden et al. proved that tobramycin DPI was expected to be more cost-effective than nebulized tobramycin by using their economic analysis [100].

Stass et al. designed a phase I clinical study with 25 patients to illustrate the tolerability and pharmacokinetic properties of ciprofloxacin DPI in CF adults with P. aeruginosa infection. It was well tolerated with minimal systemic effects compared to the oral and intravenous routes of administration [101].

These studies demonstrated the efficacy of DPI medications, and they were well designed but mainly focused on comparing the effectiveness of treatment groups to the placebo group; nevertheless, there is a lack of studies investigating the efficacy of DPIs in comparison to other routes of administration, such as DPI versus intravenous injection or DPI versus oral administration; furthermore, none of the trials’ time periods met the 12 months’ that are recommended by the European Medicines Agency for efficacy purposes; longer period research needs to be performed to give patients a full spectrum of different routes of medication’s efficacy [97].

## 8. Dry Powder Inhalers in the Pipeline

Current DPIs for treating CF in the pipeline include OligoG (Phase II) and Inhaled Mannitol (Bronchitol) (approved), both of which have the effect of improving mucociliary clearance; Colobreathe^®^ (Colistimetate sodium) (Phase III); and TOBI Podhaler^®^ (Tobramycin inhaled powder), which was approved for *P. aeruginosa* chest infection in CF.

OligoG is a dry powder drug with low molecular-weight alginate oligosaccharides that has been shown to decrease the thickness of airway mucus, which improves mucus clearance. A randomized, double-blind, placebo-controlled multicentre crossover phase 2b study was completed, which was conducted in the United Kingdom, Germany, Sweden, Denmark, and Norway, and the result indicated that inhalation of OligoG DPI over 28 days was safe in adult CF patients, although the statistically significant improvement of FEV1 was not reached [78].

The Inhaled dry powder mannitol (Bronchitol^®^) works by drawing water into the airway, thereby thinning the sticky mucus and improving mucociliary clearance. Two phase III studies of inhaled mannitol in people with CF ages 6 years and older were completed in 2011 and 2012 [38,102]. All the results show positive effects on improving lung function, and it has been approved for the treatment of CF in Australia, Europe, Russia, Israel, and the USA.

Inhaled Colistimetate sodium is an antibiotic being studied to potentially treat CF-related chronic *P. aeruginosa* infections by disrupting the bacterial outer cell membrane [93]. Schuster et al. conducted a phase III study in the UK and Germany to assess the efficacy and safety of the new dry powder formulation, the inhaled Colistimetate sodium, in CF patients over 6 years old with chronic *P. aeruginosa* pulmonary infection, and the formulation demonstrated efficacy and was well tolerated among the 380 subjects [89].

The tobramycin antibiotics kill bacteria by inhibiting bacterial protein synthesis, and their inhaled powder was approved in March 2013 by the FDA. It is the only approved inhaled antibiotic in the US. It has been reported to be safe and efficacious for treating P. aeruginosa infection in a phase III study, and after being licensed, another phase IV study was conducted in 2017 and proved that tobramycin and DPI were easy to use and performed better than traditional nebulizers [99,103].

## 9. Opportunity for Inhalable CFTR Modulators

In CFTR modulator therapy, there are two steps involved in general treatment: firstly, correcting the cellular defects that allow normal CFTR production, and secondly, the potentiation of the CFTR protein to further increase channel opening. Consequently, combination therapies of CFTR correctors and potentiators are available to CF patients, such as lumacaftor/ivacaftor, tezacaftor/ivacaftor, and elexacaftor/tezacaftor/ivacaftor, while ivacaftor is the CFTR potentiator that increases CFTR opening probability, and others are correctors that increase the amount of CFTR protein on the cell surface [63].

Since most disease mortality and morbidity are caused by respiratory manifestations, pulmonary drug delivery has the potential to enhance treatment efficacy with fewer adverse side effects than systemic administration [7].

Nevertheless, there are various possible barriers to the CFTR modulators’ DPI formulation, such as the CFTR modulators drug particles’ physicochemical properties, which should be considered before evaluating the dry powder formulation. A study performed by Garbuzenko et al. demonstrated the lipid-based nanocarrier was appropriate for the lumacaftor/ivacaftor encapsulation, and the direct pulmonary delivery showed high effectiveness in treating CF lung manifestations. Although the route of administration was not DPI, it still brings hope for the successful evaluation of CFTR modulator dry powder formulation in the future [104]. Guan et al. used an L-leucine co-amorphous formulation to enhance the ivacaftor dissolution rate and improve its aerosol performance. They report that their formulation could be a potential approach for direct pulmonary administration of ivacaftor [105].

The stability of CFTR modulators in DPI formulations is critical to their development. CFTR modulators are often sensitive to moisture and may lose potency or undergo changes in chemical properties when exposed to moisture. Therefore, careful selection of storage conditions is necessary to ensure stability and maintain drug potency in DPI formulations. Additionally, since ivacaftor has very low water solubility, its ability to penetrate the mucus layer on the airway epithelium and its impact on drug efficiency are currently unknown [106].

Ensuring dose consistency in DPI formulations is crucial for the effective delivery of CFTR modulators to patients. However, achieving dose consistency can be challenging due to several factors, such as the patient’s inhalation technique and DPI device design.

Furthermore, developing a DPI formulation for CFTR modulators must comply with regulatory standards, including demonstrating safety, efficacy, and quality. This process can be complex and may take 12–15 years to launch a finished product [107].

Furthermore, personalized CFTR modulator therapies’ also influence the DPI formulation. Personalized medicine is a term to describe a therapeutic approach that emphasizes the individualized clinical profiling of patients, taking into account the varied manifestations of symptoms, the degree of severity, and the genetic distinctions of each individual [108]. For example, the CFTR potentiator Ivacaftor is effective in treating patients with Class III CFTR mutations, whereas the CFTR corrector/potentiator combination therapy Lumacaftor/Ivacaftor is effective in treating patients with Class II CFTR mutations [109,110]. Considering the varying physicochemical properties of different drug molecules, the formulation of DPIs may be influenced, indicating that a single CFTR modulator DPI formulation may not be adequate to address the diverse spectrum of CFTR mutations.

Overall, the formulation of CFTR modulators as DPI requires meticulous attention to several key factors, such as drug particle properties, formulation stability, dose consistency, regulatory compliance, and personalized treatment. These challenges must be addressed to ensure the successful development and commercialization of DPI for CFTR modulators.

## 10. Conclusions

In conclusion, the evidence suggests that various DPI medications can be effective and safe in treating CF by improving lung function, reducing symptoms, and preventing pulmonary exacerbations, with fewer systemic side effects in comparison to traditional routes of drug administration [23,24,25]. Additionally, DPIs may also improve the patient’s adherence to treatment as they are easier and more convenient to use. However, to fully evaluate the benefits and limitations of dry powder therapy for CF treatment, further research is needed. Furthermore, most of the on-market DPIs for CF are symptomatic relievers; no DPI medications that restore CFTR function are currently available to patients.

## Figures and Tables

**Table 1:** Classifications of DPIs in respiratory disease treatment and examples [38,78,79,80,81,82,83].

Type	Treatment	Mechanism of Action	Status:
Mucus thinners	Mannitol	Mucus hydration	To patients FDA/EMA
	Alginate oligosaccharide	Macromolecules degradation	Phase II
Bronchodilators	Albuterol	Relaxation of smooth muscle	To patients FDA/EMA
	Salmeterol	Relaxation of smooth muscle	To patients FDA/EMA
	Levalbuterol	Relaxation of smooth muscle	To patients FDA/EMA
Antibiotics	Tobramycin	Inhibition of protein synthesis	To patients FDA/EMA
	Colistin	Disruption of the outer cell membrane	To patients EMA/Phase III USA
	Ciprofloxacin	Inhibition of bacterial DNA replication	Phase II

## Data Availability

The data that support the findings of this study are available from the corresponding author upon reasonable request. Some data may not be made available because of privacy or ethical restrictions.

## Abbreviations

API	Active pharmaceutical ingredient
CF	Cystic fibrosis
CFTR	Cystic fibrosis transmembrane conductance regulator
DPI	Dry powder inhaler
ENaC	Epithelium sodium channel
FEV1	Forced expiratory volume per second
IDP	Inhalable dry powder
LRT	Lower respiratory tract
*P. aeruginosa*	*Pseudomonas Aeruginosa*
SABA	short-acting beta-agonists
URT	Upper respiratory tract
